# Pulmonary Fibrosis and Progressive Pulmonary Fibrosis in a Prospective Registry of Interstitial Lung Diseases in Eastern Siberia

**DOI:** 10.3390/life13010212

**Published:** 2023-01-11

**Authors:** Maria S. Nashatyreva, Irina N. Trofimenko, Boris A. Chernyak, Sergey N. Avdeev

**Affiliations:** 1 Irkutsk State Medical Academy of Postgraduate Education–Branch Campus of the Federal State Budgetary Educational Institution of Further Professional Education “Russian Medical Academy of Continuing Professional Education”, Healthcare Ministry of the Russian Federation, mkr Yubilejnyy 100, Irkutsk 664079, Russia; 2 Federal State Autonomous Educational Institution of Higher Education I.M. Sechenov First Moscow State Medical University of the Ministry of Health of the Russian Federation, 8 Trubetskaya Str, Build. 2, Moscow 119991, Russia; 3 Federal Pulmonology Research Institute, Federal Medical and Biological Agency of Russia, Orehovyy Bul. 28, Moscow 115682, Russia

**Keywords:** interstitial lung diseases, pulmonary fibrosis, progressive pulmonary fibrosis, idiopathic pulmonary fibrosis

## Abstract

Interstitial lung diseases (ILD) are part of a large heterogeneous group of diseases that differ in many ways (in their cause, clinical presentation, and response to therapy, etc.), but there are similar pathophysiological mechanisms involved in the development of the inflammation and/or fibrosis of the lungs. Currently, several criteria for pulmonary fibrosis (PF) and progressive pulmonary fibrosis (PPF) are proposed, and the information on the prevalence and characteristics of these conditions is limited. The aim of this study was to evaluate the spectrum of PF and PPF according to the registry of patients with ILD in eastern Siberia. Materials and methods: The study included patients with ILD from all of the medical institutions in the Irkutsk region (eastern Siberia). Each case of ILD (*n* = 270) was reviewed by a multidisciplinary discussion panel. The ILD patient registry included information on the clinical findings, history, pulmonary function tests, high-resolution computed tomography (HRCT), and histological findings. The follow-up period for the patients varied from 1 to 5 years. Results: Pulmonary fibrosis was detected by HRCT in 104 patients with ILD (38.5%). PF was present in 100% of the patients with IPF and SS-ILD, in 90.9% of the patients with CHP, in 71.4% of the patients with NSIP, and in 60% of the patients with RA-ILD. Sixty-two patients met the criteria for PPF (23.0% of the entire ILD cohort and 59.6% of the patients with PF). PPF occurred most often in the patients with IPF, CHP, IPAF, and SSc-ILD: 100%, 72.7%, 40%, and 38.5% of them, respectively. The variables associated with fibrosis progression included Velcro crackles (OR 18.3, *p* < 0.001) and late diagnosis (OR 4.1, *p* < 0.001). Conclusion: Pulmonary fibrosis and progressive pulmonary fibrosis are common in patients with ILD. The high mortality rate of PPF dictates the need for the active, early detection of a progressive fibrosing course of a wide range of ILD and suggests that further studies assessing the effectiveness of the interventions might be warranted.

## 1. Introduction

Interstitial lung diseases (ILD) are a heterogeneous group of diffuse parenchymal processes in the lungs [1,2,3,4]. Currently, ILD are represented by more than 200 different entities that are heterogeneous in etiology, radiological and histological patterns, and prognosis, but there are similar pathophysiological pathways for the development of pulmonary inflammation and/or fibrosis [5,6,7].

Pulmonary fibrosis occurs in many ILD, where idiopathic pulmonary fibrosis (IPF) is a classic variant of progressive fibrosing ILD that is characterized by a rapidly progressive disease and a high mortality rate [8,9,10]. At the same time, other common forms of ILD, including idiopathic nonspecific interstitial pneumonia (iNSIP), chronic hypersensitivity pneumonitis (CHP), and connective tissue disease-associated ILD (CTD-ILD) [9,11], may also have signs of pulmonary fibrosis (PF), which as in the case of IPF can worsen or progress [12].

In progressive fibrosing ILD, high-resolution computed tomography (HRCT) reveals an increase in the amount of fibrotic changes in the lung parenchyma, a decrease in the lung function during serial observation, the worsening of symptoms, a decrease in the quality of life, and early mortality, despite conventional therapy with glucocorticosteroids and/or immunosuppressants [7,9,10]. In this regard, such variants of the course of ILD are increasingly described in the general terminology as progressive fibrosing ILD or progressive pulmonary fibrosis (PPF) [7,10,11]. The definitions of the progression of pulmonary fibrosis vary significantly, and so far, there are no universally accepted criteria [2,13,14].

A retrospective analysis of the data from a number of studies showed that in patients with IPF with a relative decrease in the forced vital capacity (FVC) of 10–15%, the risk of death was more than two times higher than it was in patients with an absolute decrease in the FVC of <5% (hazard ratio (HR) 2.20) [15]. Other studies have shown that even in the absence of a decrease in the FVC, an increase in the size of the area of the fibrotic changes according to the HRCT data is also a prognostic factor [16,17,18]. The worsening of symptoms alone or in combination with a decrease in the FVC or the progression of fibrosis in the HRCT also does not rule out the progression of ILD, but it requires further research to confirm it as a reliable predictor of the PPF outcome [1].

Relatively recently, an effective pharmacotherapy for the treatment of IPF has emerged: antifibrotics pirfenidone and nintedanib have been shown to slow the decline in lung function and may prevent the exacerbations of IPF [19]. In addition, antifibrotic agents have been shown to be effective in other ILD: nintedanib was shown to slow down the decline in the lung function in ILD associated with systemic sclerosis [14] and the progressive fibrosing ILD of various etiologies [11]. Based on these encouraging results, it is expected that a significant proportion of ILD patients with PPF may benefit from antifibrotic drugs.

Currently, there is only a little bit of information on the prevalence of PF and PPF in patients with various ILD. The current literature data are based on the results of a survey of doctors, according to which approximately 13–40% of the patients with ILD develop PPF [20,21,22,23,24]. The overall estimated prevalence of PPF, according to some studies, ranges widely from 2.2 to 20 per 100.000 people in Europe [25,26,27], and it occurs in 28 per 100.000 people in the United States [28], which most likely reflects the geographic and methodological heterogeneity used to calculate the rates rather than true differences the prevalence [29].

Therefore, we conducted a study to evaluate the spectrum of PF and PPF in a large cohort of patients with ILD based on the eastern Siberia registry. Our secondary aims included the characteristics of these conditions, including the underlying disease etiology, and also the potential factors associated with progression.

## 2. Materials and Methods

### 2.1. Study Population

Our cohort study was based on a prospective registry of patients with ILD in eastern Siberia. All of the consecutive patients over 18 years old with established or suspected diagnosis of ILD were prospectively registered in the database between December 2018 and December 2021. All of the referred ILD patients were reviewed in a multidisciplinary discussion (MDD). The multidisciplinary panel included pulmonologists, radiologists, pathologist, and whenever they were needed, rheumatologists or occupational physicians. All of the physicians involved in the multidisciplinary discussions were experienced in the management of patients with ILD. A final diagnosis was proposed when the team reached an agreement.

The study was approved by the Institutional Review Board of Irkutsk Scientific Research Branch of Russian Medical Academy of Continuing Professional Education (Protocol No. 9 of 29 November 2018). Written informed consent for participation in the study was obtained from every patient.

### 2.2. Data Collection

Data on the patient demographics, smoking habits, symptoms at the time of diagnosis, radiologic findings, pulmonary function tests, histopathologic findings, treatment regimen and duration, progression of the disease, and survival were collected by reviewing the medical records.

All of the patients underwent HRCT on 1.0–1.5-mm-thick overlapping sections using a high-spatial-frequency reconstruction algorithm, which were taken during a single breath hold using various computed tomography scanners. The HRCT images were evaluated by 2 experienced radiologists.

The PFTs were performed according to the recommendations of the Global Lung Function Initiative. The static lung volumes were measured using the plethysmography method.

The follow-up period ranged from 1 to 5 years. The frequency of the examinations was assessed individually depending on the specific form of the disease, the severity of the course, and the maintenance therapy.

### 2.3. Definitions

Pulmonary fibrosis was defined as the presence of traction bronchiectasis, reticulations with/without honeycombing with features of fibrosis affecting more than 10% of lung volume on HRCT confirmed by central review [11].

Progressive pulmonary fibrosis was defined as the patient meeting at least one the following criteria within 24 months before the screening despite treatment with corticosteroids and immunosuppressants:

(1) A relative decline in the FVC of at least 10% of the predicted value;

(2) A relative decline in the FVC of 5% to <10% of the predicted value and the worsening of the respiratory symptoms, or increased extent of fibrosis on HRCT;

(3) The worsening of the respiratory symptoms and increased extent of fibrosis on HRCT.

IPF was diagnosed according to the guideline criteria available at the time of diagnosis [30]. A diagnosis of idiopathic interstitial pneumonia (IIP) required chest HRCT with an appropriate pattern of a specific type of IIP or confirmation by surgical lung biopsy [31]. CHP was diagnosed based on the clinical history, radiographic pattern, and if it was applicable, a pathological confirmation [32]. CTD-ILD required the confirmation of an underlying CTD with clinical and immunological patterns according to the currently proposed diagnostic criteria [33,34,35]. Interstitial pneumonia with autoimmune features (IPAF) was diagnosed according to the proposed research criteria [36]. The patients with an ILD that was secondary to other causes (e.g., pneumoconiosis, etc.) were included in the analysis and grouped into a category labelled “Other” ILD.

### 2.4. Statistical Analysis

For the statistical analysis, the Statistica 12.0 program (Statsoft, Inc., Tulsa, OK, USA) was used. The data were expressed as mean and standard deviation (M ± SD) or median, lower, and upper quartiles (Me (Q1;Q3)), depending on the distribution. The normality of the distribution was checked using the Shapiro–Wilk test. A chi-square statistic test or Fisher’s exact test was used for the categorical data, and an unpaired Student’s *t* test or a Mann–Whitney test was used for continuous data. Logistic regression analyses were used to investigate the risk factors for the PPF. The adjusted odds ratio (OR) values and 95% confidence intervals (CI) are reported. A *p* value of less than 0.05 was considered to be statistically significant (two-tailed).

## 3. Results

In total, 293 patients were referred for MDD between December 2018 and December 2021, and after excluding 23 patients with infectious, oncological, and cardiogenic etiologies or insufficient data, 270 patients were enrolled in the registry. The mean age at ILD diagnosis was 58.5 (46;67) years, and women slightly predominated the group (58.5%). One hundred and six patients had ILD with a known etiology, 66 patients had IIP, 74 patients had sarcoidosis, and 24 patients had another ILD. The patients with IIP were statistically significantly older than the patients from the other groups were, with mean age 67 (61;75) years (*p* < 0.001). The functional status was significantly worse in the ILD patients with a known etiology and IIP: FVC 74.4 ± 21.9% and 78.2 ± 21.9%, respectively, while the FVC in patients with sarcoidosis was significantly higher—92.5 ± 20.7%.

**Figure 1:**
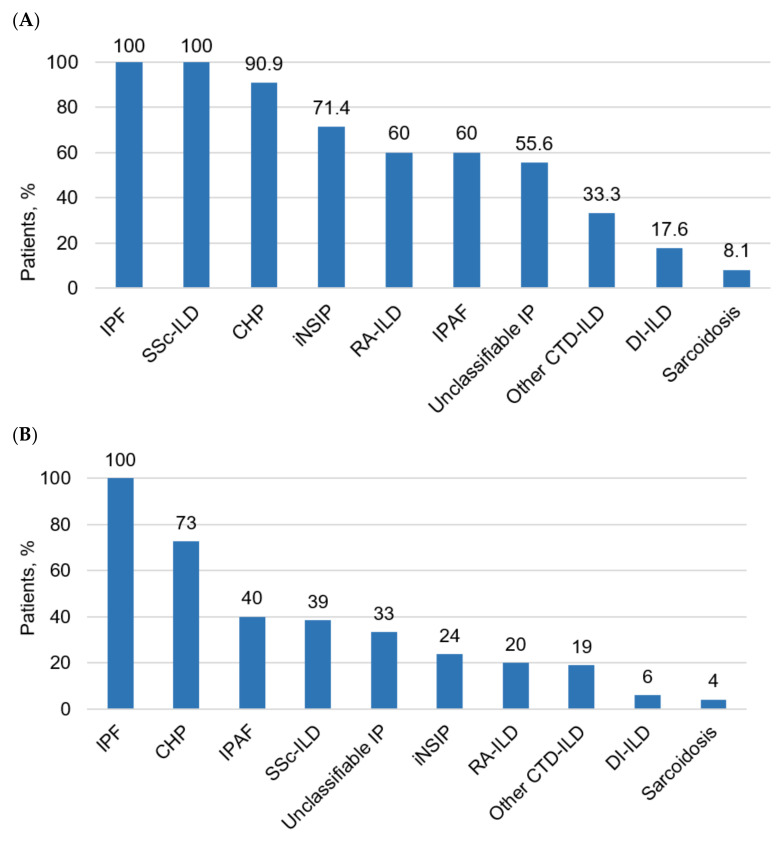
Proportion of patients with pulmonary fibrosis (**A**) and progressive pulmonary fibrosis (**B**) among different ILD. IPF—Idiopathic pulmonary fibrosis; SSc-ILD—interstitial lung disease associated with systemic sclerosis; CHP—chronic hypersensitivity pneumonitis; iNSIP—idiopathic nonspecific interstitial pneumonia; RA-ILD—rheumatoid arthritis-associated interstitial lung disease; IPAF—interstitial pneumonia with autoimmune features; IP—interstitial pneumonia; CTD-ILD—connective tissue disease-related interstitial lung disease; DI-ILD—drug-induced interstitial lung disease.

Signs of PF were detected by HRCT in 104 patients (38.5%). The largest number of patients with PF was observed among the ILD patients with a known etiology (*n* = 58; 54.7%) and in the patients with IIP (*n* = 40; 60.6%) with an absolute predominance of patients with IPF. Sixty-two patients met the criteria of PPF (23.0%) in entire ILD cohort and 59.6% of the patients had PF. PPF occurred most often in the patients with IPF, CHP, IPAF, and SSc-ILD: 100%, 72.7%, 40%, and 38.5%, respectively (Figure 1).

Among the patients with ILD, the highest proportion of PF was observed in the patients with IPF, SSc-ILD, CHP, iNSIP, and RA-ILD: 100%, 100%, 90.9%, 71.4%, and 60.0% of the cases, respectively (Table 1). In terms of sarcoidosis, only 8.1% of the patients had signs of pulmonary fibrosis, whereas in the patients with organizing pneumonia, no cases of PF were observed (Table 1).

The ILD patients with PF were older than the ILD patients without PF: 61.4 ± 13.6 years vs. 53.1 ± 15.2 years (*p* < 0.001). In general, there were no significant differences in the age, gender, symptom severity, and lung function between the PF and PPF patient groups. In the group of patients with PF, smoking was more common (46.1% vs. 31.9%; *p* = 0.018), and a history and contact with harmful occupational or domestic factors was also more common (34.6% vs. 22.9%; *p* = 0.03). In the patients with PF, clubbing was observed six times more (22.1% vs. 3.6%; respectively, *p* < 0.001), as well as Velcro crackles (80.8% vs. 18.6%; *p* < 0.001). In the ILD patients with PF, the symptoms were also significantly more common: dyspnea (96.2% vs. 68.1%; *p* < 0.001), a cough (93.3% vs. 72.3%; *p* < 0.001), weakness (78.8% vs. 63.2%; *p* = 0.007), and weight loss (31.7% vs. 16.8%; *p* = 0.007) (Table 2). The patients with PF had significantly lower PFT parameters: FVC 69 (58;87)% vs. 88 (73;101)%, predicted at *p* < 0.001.

The mortality rate from all of the causes during the observation period among the ILD patients with PF was significantly higher than it was in the patients without PF (45.2 and 11.4%, respectively; *p* < 0.001).

The clinical, functional, and HRCT characteristics of the ILD patients with PPF are presented in Table 2. Significant differences between the patients with various ILD in the PPF group were observed in clubbing (*p* = 0.05) and honeycombing (*p* < 0.001), which were more common in the patients with IPF (Table 3). In addition, the IPF patients were characterized by had the highest mortality rate (100%) and the shortest time interval from the first symptoms onset to death, 35.5 (24;49) months (*p* = 0.01), compared with those of other PPF patients (Table 3).

The heterogeneity of the PPF cohort is noteworthy. Thus, IPF and CHP with PPF do not statistically significantly differ in terms of parameters such as the severity of dyspnea (*p* = 0.31) and a cough (*p* = 0.27), SpO2 (*p* = 0.98), FVC (*p* = 0.58), honeycombing (*p* = 0.3), and the time from the appearance of the first symptom to diagnosis (*p* = 0.50) (Table 3). However, the time from the onset of the disease to death in the patients with CHP and PPF was longer than it was in IPF (*p* = 0.042).

Compared to IPF, CTD-ILD with PPF were characterized by a more favorable course in terms of the parameters such as the severity of dyspnea (*p* = 0.009) and a cough (*p* = 0.009), the presence of clubbing (*p* = 0.03), SpO2 (*p* = 0.009), and mortality (*p* = 0.005).

Risk factors for PPF among the patients with ILD based on the logistic regression analysis are presented in Table 4. The greatest contribution was made by the factors such as Velcro crackles, odds ratio (OR) 18.3 (*p* < 0.001), and a late diagnosis, OR 4.1 (*p* < 0.001) (Table 4).

## 4. Discussion

In this study, we evaluated the actual prevalence of PF and PPF in a large cohort of ILD patients using objective criteria based on PFT and HRCT. Pulmonary fibrosis was detected in 104 of ILD patients (38.5%). The most frequent PF diagnoses were IPF (100%), SSc-ILD (100%), CHP (90.9%), iNSIP (71.4%), and RA-ILD (60%). Sixty-two patients met the criteria for PPF (23.0% of the entire ILD cohort and 59.6% of the patients with PF). PPF occurred most often in the patients with IPF (100%), CHP (72.7%), IPAF (40%), and SSc-ILD (38.5%), respectively.

The literature currently presents several studies on the prevalence of fibrosing ILD, which ranges from 16.34 per 100.000 of the population [25] to 30.3 per 100.000 among men and 27.5 per 100.000 among women [28]. The prevalence of pulmonary fibrosis according to our registry was 21.8 per 100.000 population, while the prevalence of PPF is 13.0 per 100.000 population, which is comparable with the epidemiological data presented in the literature.

Most often, PF and PPF in ILD were observed in the patients with IIP (60.6% and 39.4%, respectively) and somewhat less often in the patients with a known etiology of ILD (54.7% and 31.1%, respectively), while only rare cases of PF and PPF were recorded among the patients with sarcoidosis (8.1% and 4.1%, respectively). However, when we were evaluating various ILD forms, it was found that with the exclusion of IPF, which is characterized by a 100% progressive fibrosing course, PPF was most often presented in CHP (72.7%). The data of the INBUILD study also indicate the high prevalence of CHP among PPF [11]. In addition, the results of a comparative study of patients with IPF and CHP with PPF demonstrate a comparable profile of the disease progression and the survival of patients with these ILD [37].

In a recently published study based on the data from Canadian Registry for Pulmonary Fibrosis from between 2015 and 2020, Hambly et al. reported that 1376 out of 2746 patients (50%) met the PPF criteria [38]. PPF occurred in 427 (59%) patients with IPF, 125 patients (58%) with fibrotic CHP, 281 patients (51%) with unclassifiable ILD (U-ILD), and 402 patients (45%) with CTD-ILD. Compared with IPF, the time to progression was similar in the patients with CHP, but it was delayed in the patients with U-ILD and CTD-ILD.

The logistic regression analysis of our data showed that bilateral Velcro crackles was the most significant risk factor for PPF: OR 18.3 (95% CI 9.8–34.2, *p* < 0.001). In a study of 132 patients with suspected ILD, bilateral Velcro crackles were noted in 63% of the patients, and they were associated with an HRCT pattern of usual interstitial pneumonia [39]. Thus, in the patients with ILD, Velcro crackles should be considered as an alarming factor for the progressive fibrosing course of the disease. In addition to Velcro crackles, the significant risk factors for PPF were a late diagnosis of ILD, OR 4.1 (95% CI 2.4–7.1, *p* < 0.001), as well as dyspnea at the onset of the disease, weight loss, being male, being over 65 years old, and a having history of smoking.

In our study the proportion of progressive autoimmune ILD was 24.1%, among which more than 2/3 of the patients are represented, as in the INBUILD study [11], by two diseases—SSc and RA. Similar data were observed in the study by Atienza-Mateo et al. Within the group of 111 CTD-ILD patients, most of the cases had a diagnosis of RA (27.0%), SSc (26.1%), or anti-synthetase syndrome (17.1%) [40]. There were significantly smaller proportions of the patients with iNSIP (8.2% of all patients with PPF) and unclassifiable IIP (4.9%) than those in the INBUILD study.

It should be noted that IPF, as a prototype of PPF, was characterized by the most unfavorable prognosis among all of the patients with PF. Thus, the median time from the first symptom to death was 35.5 months, which corresponds with the literature data [41,42]. The high mortality rate among the patients with PPF requires the more active monitoring of patients with fibrosing ILD in order to timely determine the disease progression and the necessary change in the therapeutic tactics.

Our study had some limitations. Firstly, our study is based on data from only one regional registry. The patients in our registry were recruited from several tertiary care centers, and thus, it is possible that referral bias could also lead to an overestimation of the prevalence of PF and PPF. Secondly, we used the INBILD criteria, and the evidence of progression was only assessed up to 24 months after the diagnosis, and so extending follow-up over time could lead to an increased prevalence of meeting the PPF criteria over time. Thirdly, in our study, among the patients with PF, the proportion of patients with IPF was only 10%. This fact is rather difficult to explain, and we can only speculate that the geographic location, the relatively young age of the population of our region, and the features of the industrial infrastructure, etc., contribute to this. On the other hand, a relatively low proportion of patients with IPF among the ILD cases was observed in certain regions, for example, in a study from India, the overall proportion of IPF was 13.7% [43], and in a study from the greater Paris region, it was 11.6% [25]. Fourthly, in our study we did not assess the effect of the antifibrotic therapy on the outcomes because the study began before the approval of antifibrotics for PPF, but this factor should be taken into account in future studies.

## 5. Conclusions

Pulmonary fibrosis and progressive pulmonary fibrosis are common in patients with ILD. The high mortality rate of PPF dictates the need for the active, early detection of a progressive fibrosing course of a wide range of ILD and suggests that further studies assessing the effectiveness of interventions might be warranted.

## Figures and Tables

**Table 1:** Spectrum of interstitial lung diseases according to the ILD registry.

ILD Variants	ILD without PF, *n* (%)	ILD with PF, *n* (%)	ILD with PPF, *n* (%)
All ILD	166 (61.5)	104 (38.5)	62 (23.0)
ILD with known etiology	38 (40.9)	55 (59.1)	30 (32.3)
CHP	2 (9.1)	20 (90.9)	16 (72.7)
CTD-ILD	22 (40.7)	32 (59.3)	13 (24.1)
SSc-ILD	0	13 (100.0)	5 (38.5)
RA-ILD	8 (40.0)	12 (60.0)	4 (20.0)
Other autoimmune ILD ^#^	14 (66.7)	7 (33.3)	4 (19.0)
DI-ILD	14 (82.4)	3 (17.6)	1 (5.9)
IIP (total)	24 (38.1)	39 (61.9)	26 (41.3)
IPF	0	16 (100.0)	16 (100.0)
iNSIP	6 (28.6)	15 (71.4)	5 (23.8)
Unclassifiable IP	4 (44.4)	5 (55.6)	3 (33.3)
IPAF	2 (40.0)	3 (60.0)	2 (40.0)
COP	12 (100.0)	0	0
Sarcoidosis	68 (91.9)	6 (8.1)	3 (4.1)
Rare ILD	22 (100.0)	0	0
ICEP	9 (100.0)	0	0
LCH	5 (100.0)	0	0
LAM	5 (100.0)	0	0
PAP	3 (100.0)	0	0
Other ILD *	14 (77.8)	4 (22.2)	3 (16.7)

ILD—interstitial lung diseases; PF—pulmonary fibrosis; PPF—progressive pulmonary fibrosis; CHP—chronic hypersensitive pneumonitis; SSc—systemic sclerosis; RA—rheumatoid arthritis; DI-ILD—drug-induced ILD; IIP—idiopathic interstitial pneumonia; IPF—idiopathic pulmonary fibrosis; iNSIP—idiopathic nonspecific interstitial pneumonia; IP—interstitial pneumonia; IPAF—interstitial pneumonia with autoimmune features; COP—cryptogenic organizing pneumonia; ICEP—idiopathic chronic eosinophilic pneumonia; LCH—langerhans cell histiocytosis; LAM—lymphangioleiomyomatosis; PAP—pulmonary alveolar proteinosis; # ILD, associated with ankylosing spondylitis, mixed connective tissue disease, systemic lupus erythematosus, Sjogren ‘s disease, dermatopolymiositis, and vasculitis. * other ILD included pneumoconiosis, ILD associated with HIV, lymphocytic interstitial pneumonia in patient with general variable immune insufficiency, ILD associated with nonspecific ulcerative colitis, lymphoid interstitial pneumonia, respiratory bronchiolitis-associated interstitial lung disease, pulmonary amyloidosis, and pulmonary alveolar microlithiasis.

**Table 2:** Clinical, functional, and HRCT characteristics of ILD patients without PF and with PF and PPF.

Value	ILD without PF	ILD with PF	ILD with PPF	*p* ^#^	*p* ^##^
Number, *n*	166	104	62		
Age, years	55 (42;65)	64 (55;70)	64 (56;73)	<0.001	0.71
Male, *n* (%)	59 (35.5)	53 (51.0)	36 (58.1)	0.01	0.32
Symptoms					
Dyspnea, *n* (%)	113 (68.1)	100 (96.2)	62 (100.0)	<0.001	0.96
mMRC dyspnea, points	2 (1;2)	3 (2;3)	3 (2;3)	<0.001	0.79
Cough, *n* (%)	120 (72.3)	97 (93.3)	59 (95.2)	<0.001	0.91
Weight loss, *n* (%)	28 (16.8)	33 (31.7)	27 (43.5)	0.004	0.10
Clubbing, *n* (%)	6 (3.6)	23 (22.1)	20 (32.2)	<0.001	0.10
Velcro crackles, *n* (%)	31 (18.6)	84 (80.8)	53 (85.5)	<0.001	0.13
Functional characteristics					
SpO2, %	97 (95;98)	95 (92;97)	94 (92;96)	<0.001	0.31
FVC, % predicted	88 (73;101)	69 (58;87)	63 (52;76)	<0.001	0.08
HRCT patterns					
Honeycombing, *n* (%)	0	57 (54.8)	37 (59.7)	<0.001	0.74
Traction bronchiectasis, *n* (%)	0	69 (63.5)	43 (69.4)	<0.001	0.67
Reticular changes, *n* (%)	75 (45.2)	99 (95.2)	59 (95.2)	<0.001	0.96
Ground-glass opacity, *n* (%)	58 (34.9)	40 (38.5)	23 (37.1)	0.56	0.93
Consolidation, *n* (%)	51 (30.7)	13 (12.5)	9 (14.5)	<0.001	0.89
Basal predominance, *n* (%)	28 (16.9)	63 (60.6)	39 (62.9)	<0.001	0.88
Course and outcome					
Time from the onset of symptoms to diagnosis, months	6 (2;24)	24 (7;48)	30 (12;40)	<0.001	0.88
Time from the first symptom to death, months	47 (26;65)	48 (27;72)	40 (21;62)	0.62	0.55
Death, *n* (%)	19 (11.4)	47 (45.2)	37 (59.8)	<0.001	0.05

ILD—interstitial lung diseases; PF—pulmonary fibrosis; PPF—progressive pulmonary fibrosis; mMRC—modified Medical Research Council; SpO2—oxygen saturation of the blood; FVC—forced vital capacity; HRCT—high-resolution computed tomography. ^#^ *p* values compare ILD without PF and with PF. ^##^ *p* values compare ILD with PF and with PPF.

**Table 3:** Clinical, functional, and HRCT characteristics of ILD patients with PPF.

Value	IPF	CHP	CTD-ILD	Other ILD	*p*
Number, *n*	16	16	13	10	
Symptoms					
Dyspnea, *n* (%)	16 (100.0)	16 (100.0)	13 (100.0)	10 (100.0)	-
mMRC dyspnea, points	3.7 ± 0.4	3.4 ± 0.6	3.1 ± 0.5	3.4 ± 0.5	0.06
Cough, *n* (%)	16 (100.0)	16 (100.0)	13 (100.0)	10 (100.0)	-
Weight loss, *n* (%)	13 (81.2)	11 (68.7)	7 (53.8)	7 (70.0)	0.3
Clubbing, *n* (%)	10 (62.5)	9 (56.2)	3 (23.1)	4 (40.0)	0.05
Velcro crackles, *n* (%)	16 (100.0)	16 (100.0)	11 (84.6)	8 (80.0)	0.12
Functional characteristics					
SpO2, %	84.2 ± 5.5	84 ± 6.8	90.1 ± 5.3	86.5 ± 6.7	0.05
FVC, % predicted	54.7 ± 8.6	49.7 ± 15.6	59.9 ± 17.2	61.6 ± 11.9	0.13
HRCT patterns					
Honeycombing, *n* (%)	13 (81.2)	13 (81.2)	6 (46.1)	7 (70.0)	0.19
Traction bronchiectasis, *n* (%)	16 (100.0)	8 (50.0)	11 (84.6)	9 (90.0)	<0.001
Reticular changes, *n* (%)	16 (100.0)	18 (100.0)	13 (100.0)	10 (100.0)	-
Ground-glass opacity, *n* (%)	1 (6.2)	6 (37.5)	5 (38.5)	3 (40.0)	0.25
Consolidation, *n* (%)	0	0	1 (7.7)	2 (20.0)	0.04
Basal predominance, *n* (%)	14 (87.5)	9 (56.2)	8 (61.5)	3 (30.0)	0.04
Course and outcome					
Time from the onset of symptoms to diagnosis, months.	16 (6;24)	12 (9.5;48)	31 (9;84)	30 (12;48)	0.1
Time from the first symptom to death, months.	35 (24;49)	55 (23;90)	54 (27;57)	83 (36;99)	0.01
Death, *n* (%)	16 (100.0)	6 (37.5)	5 (38.5)	7 (70.0)	0.007

IPF—idiopathic pulmonary fibrosis; CHP—chronic hypersensitive pneumonitis; ILD—interstitial lung diseases; CTD-ILD—connective tissue disease-related interstitial lung disease; VAS—visual analogue scale; mMRC—modified Medical Research Council; SpO2—oxygen saturation of the blood; FVC—forced vital capacity; HRCT—high-resolution computed tomography.

**Table 4:** Risk factors of the PPF in ILD.

Feature	OR	95% CI	*p*
Velcro crackles	18.3	9.8–34.2	<0.001
Time to diagnosis ≥ 12 months	4.1	2.4–7.1	<0.001
Dyspnea at the onset of the disease	2.2	1.3–3.8	0.002
Weight loss	2.2	1.2–3.9	0.006
Age ≥ 65 years	2.1	1.2–3.3	0.005
Male gender	2.1	1.2–3.3	0.004
Current smoking	1.8	1.1–3.0	0.01

## Data Availability

Not applicable.
